# Simultaneous Detection and Estimation of Catechol, Hydroquinone, and Resorcinol in Binary and Ternary Mixtures Using Electrochemical Techniques

**DOI:** 10.1155/2015/862979

**Published:** 2015-12-01

**Authors:** Md. Uzzal Hossain, Md. Toufiqur Rahman, Md. Qamrul Ehsan

**Affiliations:** Department of Chemistry, University of Dhaka, Dhaka 1000, Bangladesh

## Abstract

Cyclic voltammetry (CV) and differential pulse voltammetry (DPV) were performed with a glassy carbon electrode (GCE) modified with polyglutamic acid (PGA) on the three dihydroxybenzene isomers, catechol (CT), hydroquinone (HQ), and resorcinol (RS). At bare GCE, these isomers exhibited voltammograms with highly overlapped redox peaks that impeded their simultaneous detection in binary and ternary mixtures. On the contrary, at PGA modified GCE binary and ternary mixtures of the dihydroxybenzene isomers showed well-resolved redox peaks in both CV and DPV experiments. This resolving ability of PGA modified GCE proves its potential to be exploited as an electrochemical sensor for the simultaneous detection of these isomers.

## 1. Introduction

The isomers of dihydroxybenzene catechol (1,2-dihydroxybenzene, CT), hydroquinone (1,4-dihydroxybenzene, HQ), and resorcinol (1,3-dihydroxybenzene, RS) are widely used in medicines, pesticides, cosmetics, tanning, flavoring agents, antioxidants, dyes, and photography chemicals [1, 2]. Due to their high toxicity and low degradability in the ecological environment, they are considered as environmental pollutants [3, 4]. Moreover, these isomers are often coexisting in environmental samples [5, 6] and interfere with each other during their identification [7]. Therefore, it is necessary to develop simple and rapid analytical method for the detection and determination of these isomers in a mixture.

Many analytical methods have been established to determine dihydroxybenzene isomers, such as HPLC [8], fluorescence [9], chemiluminescence [10], spectrophotometry [11], GC-MS [12], and electrochromatography [13]. Among them, electrochemical methods have attracted ever-growing attention due to the advantages such as fast response, low cost, simple operation, faster analysis, high sensitivity, and excellent selectivity [14]. At ordinary bare electrodes, these isomers show highly overlapping voltammograms where their redox peaks are not resolved [15–20]. Detection and estimations of CT and HQ in a binary mixture have been reported in literature [21–28]. However, the simultaneous determination of CT, RS, and HQ in a ternary mixture by electrochemical methods is insufficient. Recently, though the simultaneous determinations of CT, HQ, and RS have been performed by single-wall carbon nanotube (SWCNT) [29], modified glassy carbon electrode (GCE), modified multielectrode array [30], and graphite doped carbon ionic liquid electrode [25], it is still worthwhile to investigate novel electrode material for the simultaneous determination of CT, HQ, and RS in ternary mixture. In this paper, an effective and practical method for the simultaneous detections and quantitative estimation of CT, HQ, and RS using CV and DPV techniques at PGA modified GCE is presented.

## 2. Experimental Section

### 2.1. Reagents and Apparatus

Chemicals used in this study are (i) catechol (BDH); (ii) hydroquinone (BDH); (iii) resorcinol (BDH); (iv) NaOH pellets (Merck, Germany); (v) potassium chloride (Merck, Germany); (vi) potassium phosphate (monobasic) (Merck, Germany); (vii) L-glutamic acid (viii); sodium acetate (Merck, Germany); and (ix) acetic acid (Sigma-Aldrich). Deionized water was used for solution preparation and cleaning purposes. Solutions were purged with 99.997% dry nitrogen (BOC, Bangladesh) to remove dissolved oxygen and to maintain inert atmosphere prior to experiment. All reagents were obtained as AR grade and used without further purification.

This study was carried out using an Epsilon Electroanalyser developed by Bioanalytical System, Inc., USA, in a Pyrex glass microcell with Teflon cap. A glassy carbon electrode (GCE) was used as working electrode while Ag/AgCl and Pt wire functioned as the reference and counter electrodes, respectively. The pH of solutions was measured using Orion 2 Star (made by Thermo Electron Corporation) pH meter.

### 2.2. Electrode Modification

The bare glassy carbon electrode was first polished on polishing cloth with 0.3 *μ*m alumina and then washed with distilled water and sonicated in ethanol. The cleaned and polished GCE was placed in 0.01 M glutamic acid solution in a pH 7.0 phosphate buffer, which was previously purged with high purity nitrogen for 10 minutes. The electrode was treated with four cycles of CV between −1.5 and 2.0 V at a scan rate of 100 mV/s. A uniform adherent blue polymeric layer was observed on the electrode surface. The electrode was ready for the experiment after a rinse with deionized water. Voltammograms correspond to 1.0 mmol·L^−1^ of analyte at 50 mV·s^−1^ scan rate unless otherwise mentioned.

## 3. Results and Discussion

### 3.1. Behavior of Dihydroxybenzenes at Bare and Modified GCE

**Figure 1:**
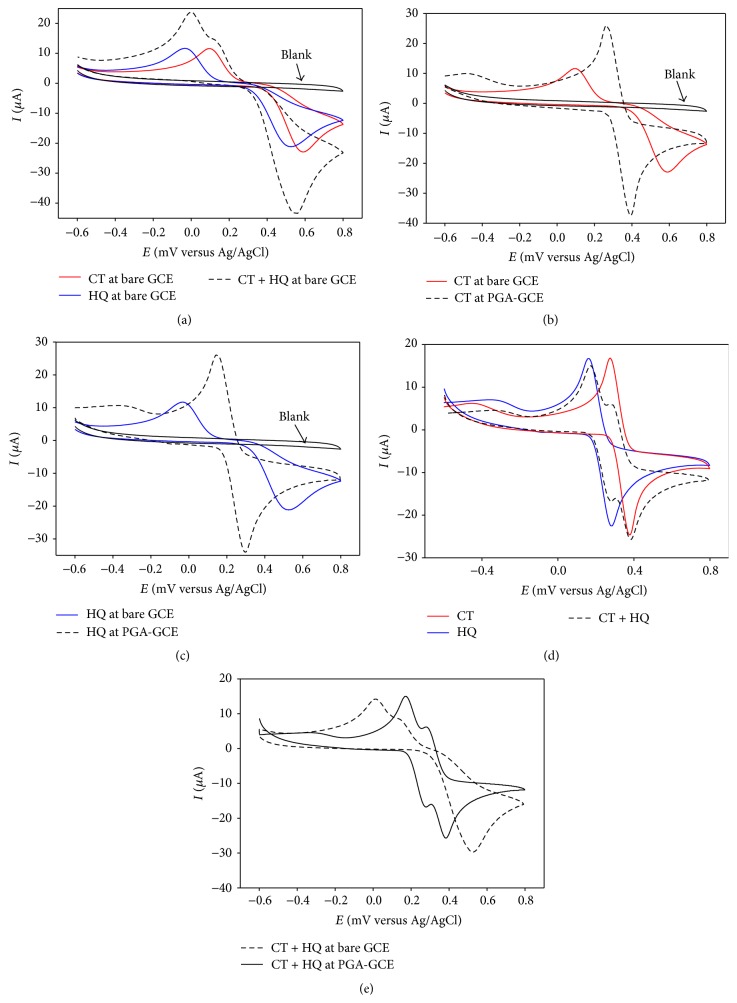
CVs of (a) CT and HQ (solid lines), CT-HQ binary mixture (dashed line) at bare GCE; (b) CT at bare GCE (solid line) and at PGA-GCE (dashed line); (c) HQ at bare GCE (solid line) and at PGA-GCE (dashed line); (d) overlay of individual CT and HQ (solid lines) and CT-HQ binary mixture (dashed line) at PGA-GCE; (e) comparison between simultaneous CT and HQ at bare GCE (dashed) and at PGA-GCE (solid line) in ABS pH 4.5.

At bare GCE in acetate buffer solution (ABS, pH 4.5) CT and HQ showed highly overlapping character where their anodic and cathodic peaks merged forming an overall voltammogram that impeded the simultaneous detection using CV at bare GCE in a binary mixture (Figure 1(a)). We observed similar behavior in UV-Vis spectroscopy. CT and HQ showed maximum absorbance at 275.45 nm and 288.60 nm wavelengths, respectively, in ABS. But in their binary mixture only one peak was observed at 278.20 nm which is the combined absorbance of catechol and hydroquinone. On the other hand at PGA modified GCE, both CT and HQ showed highly reversible behavior (ipa/ipc close to unity) in CV with dramatic signal enhancement compared to that at bare GCE (Figures 1(b) and 1(c)).

Figure 1(d) shows the CV of CT-HQ binary mixture at PGA-GCE overlaid with that of individual CT and HQ. It is clear that both CT and HQ retained their corresponding redox peaks in the mixture. A comparison between the CV responses of the binary mixture of CT and HQ at bare and PGA modified GCE is depicted in Figure 1(e). Similar behavior was observed in phosphate buffer solution (PBS, pH 7.0).

### 3.2. Electrochemical Behavior of Dihydroxybenzenes in Binary Mixtures at the Modified GCE

A series of CV and DPV experiments were performed on binary mixtures of CT, HQ, and RS in PBS pH 7.0 for their simultaneous detection in the presence of another.

#### 3.2.1. Detection of CT and HQ in Mixture in PBS

**Figure 2:**
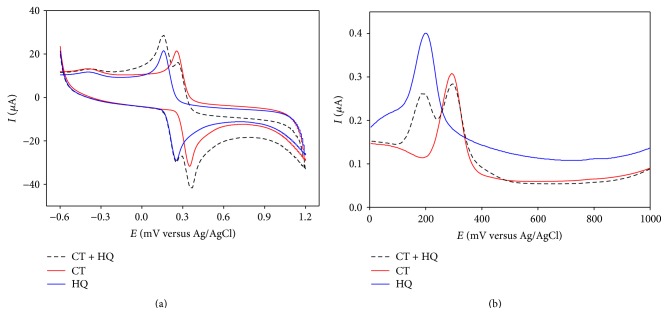
Overlay of (a) CV and (b) DPV responses of individual (solid lines) and binary mixture (dashed line) of CT and HQ in PBS at PGA modified GCE.

At PGA modified GCE, CT and HQ showed well separated anodic peaks at +252 mV and +364 mV and two cathodic peaks at +264 mV and +151 mV, respectively. An overlay of CVs of individual CT and HQ with that of CT-HQ binary mixture (Figure 2(a)) shows that both CT and HQ retain their corresponding redox peaks in the binary mixture at the modified GCE.

A DPV of the CT-HQ binary mixture in PBS is shown in Figure 2(b), which clearly shows that CT and HQ response with well separated anodic peaks while retaining their individual peak positions (at +296 mV and +193 mV, resp.) at the modified GCE. The peak separation is measured to be 103 mV that is quite good for the simultaneous detection of these isomers in the presence of another.

#### 3.2.2. Detection of CT and RS in Mixture in PBS

**Figure 3:**
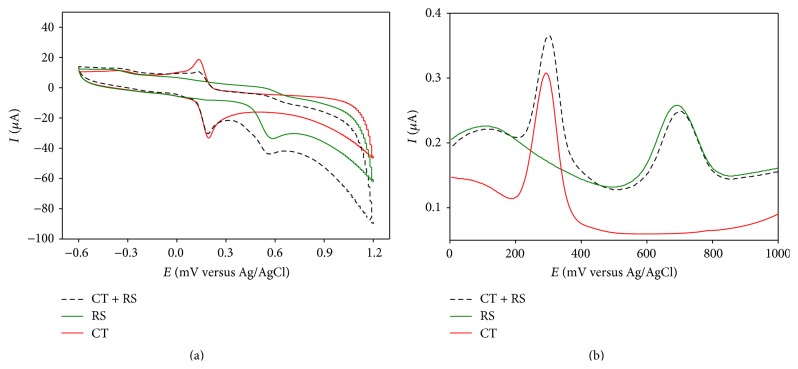
Overlay of (a) CV and (b) DPV responses of individual (solid lines) and mixture (dashed line) of CT and RS in PBS at PGA modified GCE.

Similar experiments were done on CT-RS binary mixture. In CV, CT and RS gave responses with large peak separation that was retained in the binary mixture (Figure 3(a)).

In DPV, CT and RS showed oxidation peaks (at +293 mV and +702 mV) separated by 409 mV that is well suited for their simultaneous detection at PGA modified GCE (Figure 2(b)).

#### 3.2.3. Detection of HQ and RS in Mixture in PBS

**Figure 4:**
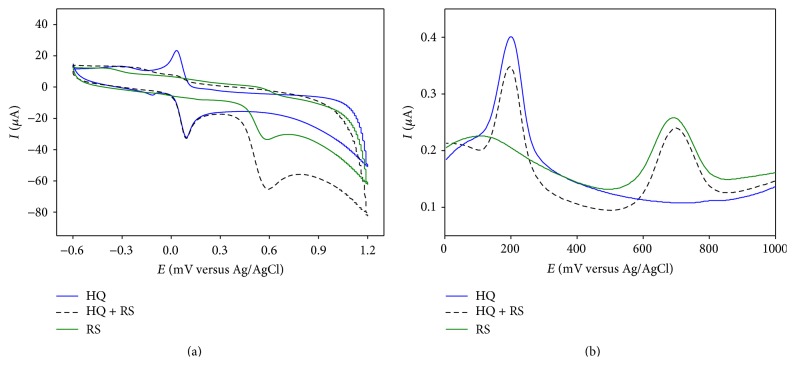
Overlay of (a) CV and (b) DPV responses of individual (solid lines) and mixture (dashed line) of HQ and RS in PBS at PGA modified GCE.

Same experiments were performed on HQ-RS binary mixture that exhibited even better peak separation in both CV and DPV depicted in Figures 4(a) and 4(b), respectively. In DPV, the binary mixture shows two anodic peaks at +202 mV and +701 mV, respectively, resulting in peak separation of 499 mV.

### 3.3. Detection of CT, HQ, and RS in Ternary Mixture

CV and DPV experiments were performed to detect CT, HQ, and RS simultaneously from ternary mixtures in both acetate buffer (ABS) and phosphate buffer solution (PBS) at PGA modified GCE.

#### 3.3.1. Detection of CT, HQ, and RS in Ternary Mixture in PBS

**Figure 5:**
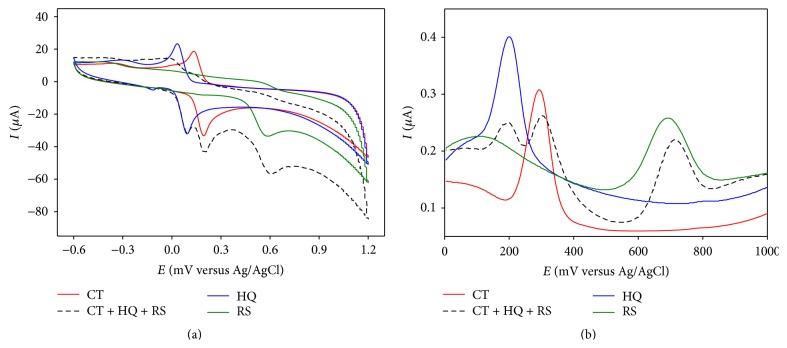
Overlay of (a) CV and (b) DPV responses of individual (solid lines) and ternary mixture (dashed line) of CT, HQ, and RS in PBS at PGA modified GCE.

Cyclic voltammograms of individual CT, HQ, and RS and that of the mixture in PBS at PGA-GCE are overlaid on Figure 5(a). Three anodic peaks at +94 mV, +201 mV, and +605 mV and two cathodic peaks at +134 mV and +19 mV were observed. These peaks correspond to the anodic and cathodic peaks of CT, HQ, and RS.

The DPV study of CT, HQ, and RS as individual isomers and as a mixture is shown in Figure 5(b), which shows that at PGA modified GCE, CT, HQ, and RS retained their corresponding anodic peaks at +295 mV, +202 mV, and +715 mV, respectively, in ternary mixture.

### 3.4. Detection of CT, HQ, and RS in Ternary Mixture in ABS

**Figure 6:**
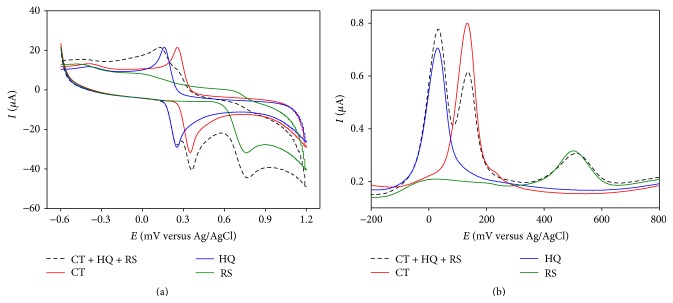
(a) CVs of individual (solid lines) and ternary mixture (dashed line) of CT, HQ, and RS; (b) DPV responses of individual (solid lines) and ternary mixture (dashed line) of CT, HQ, and RS in ABS at PGA modified GCE.

The CVs of the ternary mixture and individual CT, HQ, and RS in acetate buffer solution at PGA modified GCE were taken and are shown in Figure 6(a). Three anodic peaks at +247 mV, +354 mV, and +769 mV for the three isomers and two cathodic peaks at +224 mV and +135 mV for CT and HQ, respectively, were found.

Figure 6(b) shows the overlay of DPV responses of individual CT, HQ, and RS (at +138 mV, +34 mV, and +513 mV resp.) and that of ternary mixture in ABS.

### 3.5. Quantitative Estimation

#### 3.5.1. Quantitative Estimation of CT in the Presence of HQ

**Figure 7:**
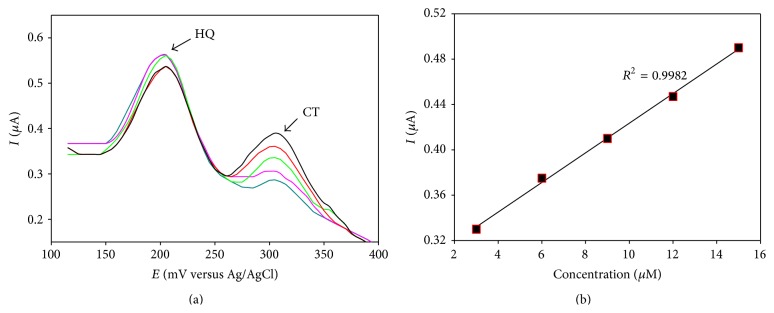
(a) Differential pulse voltammogram (DPV) of CT in the presence of HQ at PGA modified GCE in PBS. (b) Calibration curve for the quantitative determination of CT in the presence of HQ.

DPV was performed on the binary mixture of catechol and hydroquinone at PGA modified GCE within the potential range of +100 mV to +400 mV. 10.0 mL of analyte solutions was prepared to vary the amount of 1.0 mmol·L^−1^ solution CT with successive increment of 10.0 *μ*L using a micropipette while keeping HQ concentrations constant at 1.0 mmol·L^−1^. The resulting DPVs are shown in Figure 7(a).

A calibration curve (Figure 7(b)) was drawn for different concentrations of catechol. This calibration curve can be used to quantify catechol (CT) in the presence of hydroquinone (HQ) in a binary mixture. The detection limit of catechol in presence of hydroquinone was found in micromolar range.

#### 3.5.2. Quantitative Estimation of HQ in the Presence of CT and RS

**Figure 8:**
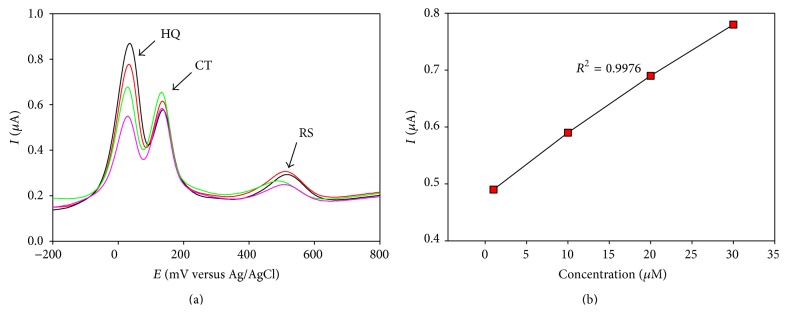
(a) Differential pulse voltammogram (DPV) HQ in the presence of CT and RS PGA modified GCE in PBS. (b) Calibration curve for the quantitative determination of HQ in ternary mixture with CT and RS.

DPV was performed on a ternary mixture of CT and HQ and RS at PGA modified GCE in the potential range of −200 mV to +800 mV. 10.0 mL of analyte solutions was prepared to vary the amount of 1.0 mmol·L^−1^ solution HQ with successive increment of 10.0 *μ*L using a micropipette while keeping CT and RS concentrations constant at 1.0 mmol·L^−1^. The resulting DPVs are shown in Figure 8(a). A calibration curve (Figure 8(b)) was drawn for different concentrations of HQ which can be used for its quantitative estimation in the presence of CT and RS. The detection limit of HQ in the presence of other isomers was found in micromolar range.

This separating ability of the polyglutamic acid modified glassy carbon electrode can be used to estimate catechol, hydroquinone, and resorcinol quantitatively in presence of others.

## 4. Conclusion

In summary, a simple yet effective method for the simultaneous detection and estimation of catechol, hydroquinone, and resorcinol using electrochemical techniques has been presented. Catechol, hydroquinone, and resorcinol have been detected from binary and ternary mixtures in ABS and PBS at PGA modified GCE. This method could be used to detect and quantify dihydroxybenzenes as pollutants and contaminants in real environmental samples. Though only qualitative studies could be done at this point, further studies towards the improvement of detection and quantitative estimation is required and will be reported in due course.
